# Severe Pneumonia Caused by *Legionella pneumophila* Serogroup 11, Italy

**DOI:** 10.3201/eid1811.120216

**Published:** 2012-11

**Authors:** Antonella Grottola, Fabio Forghieri, Marisa Meacci, Anna Fabio, Lorena Pozzi, Patrizia Marchegiano, Mauro Codeluppi, Monica Morselli, Leonardo Potenza, Ambra Paolini, Valeria Coluccio, Mario Luppi, Fabio Rumpianesi, Monica Pecorari

**Affiliations:** University of Modena and Reggio Emilia, Modena, Italy; and University Hospital-Policlinico, Modena

**Keywords:** *Legionella pneumophila*, serogroup 11, pneumonia, severe pneumonia, Italy, bacteria

**To the Editor:** *Legionella pneumophila* serogroups (SGs) 1–16 cause pneumonia in humans. Although SG 1 is the serogroup most commonly associated with disease (*1*), we report a case of community-acquired legionellosis caused by SG 11.

In November 2010, a 42-year-old man was admitted to Modena University Hospital, Modena, Italy, with a 4-day history of fever, dyspnea, and cough. His vital signs were as follows: temperature 40.0°C, pulse 135 beats/min, blood pressure 110/60 mm Hg, respiratory rate 30 breaths/min, and oxygen saturation 85% in room air. Inspiratory crackles were heard in the left lower lung lobe. Chest radiographs and successive high-resolution computerized tomography revealed left lobar infiltrates (Figure, panels A and B). Blood count documented severe pancytopenia together with high levels of inflammation markers: fibrinogen (1,031 mg/dL), C-reactive protein (33 mg/dL), and procalcitonin (28.5 ng/mL). The patient’s medical history was unremarkable; however, results of tests conducted at the time of hospital admission led to the diagnosis of acute leukemia.

**Figure:**
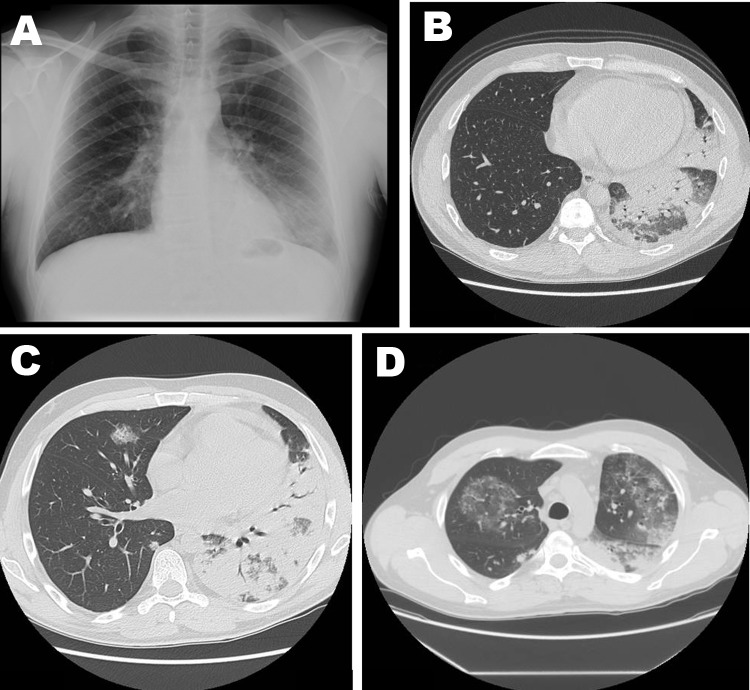
Imaging studies of 42-year-old man with severe pneumonia caused by *Legionella* *pneumophila* serogroup 11, showing lobar consolidation of the left lower lung lobe, with an air-bronchogram within the homogeneous airspace consolidation. Consensual mild pleural effusion was documented by a chest radiograph (A) and high-resolution computed tomography (B). A week after hospital admission, repeat high-resolution computerized tomography of the chest showed extensive and homogeneous consolidation of left upper and lower lobes, accompanied by bilateral ground-glass opacities (C and D).

Empirically prescribed antimicrobial treatment for neutropenic patients was initiated and consisted of meropenem (3 g/day) and levofloxacin (500 mg/day), combined first with vancomycin (2 g/day) and later with linezolid (1,200 mg/day). A few days later, antifungal therapy was empirically added to the treatment regimen (liposomal amphotericin B at 3 mg/kg/day). The patient received continuous positive airway pressure, which resulted in progressive improvement of blood gas exchange, until normalization was achieved.

Serologic and molecular examination and culture of bronchoalveolar lavage fluid, blood, urine, and feces produced negative results for fungal, viral, and bacterial pathogens. Test results for *L.* *pneumophila* urinary antigen (Biotest AG, Dreieich, Germany) and IgM and IgG against *L.* *pneumophila* (Serion-Immundiagnostica GmbH, Würzburg, Germany) were negative. Culture of sputum collected at the time of hospital admission showed growth of legionella-like colonies on buffered charcoal yeast extract, with and without the addition of antimicrobial drugs (Oxoid, Basingstoke, UK). The colonies were identified as *L. pneumophila* SGs 2–14 by the *Legionella* latex test (Oxoid). The strain was further characterized as *L. pneumophila* SG 11, according to a polyclonal latex reagent set (Biolife, Milan, Italy). Environmental investigations were conducted in the patient’s house and workplace, but *L. pneumophila* SG 11 was not detected in any of the locations tested.

A week after hospital admission, the patient was persistently febrile and experienced pain in the left thorax. High-resolution computerized tomography of the chest was repeated and showed increased pulmonary infiltrate (Figure, panels C and D) that was consistent with *L. pneumophila* pneumonia (*2*). Highly potent antimicrobial therapy against *L.* *pneumophila* was administered, consisting of high-dosage levofloxacin (1 g/day) combined with azithromycin (500 mg/day), while the other antimicrobial agents were progressively reduced (*3*). The fever subsided 14 days after the onset of targeted antimicrobial drug treatment; at that time, the sputum culture and test results for urinary *L.* *pneumophila* antigen were negative, but serologic assay results were positive for IgG and negative for IgM against *L. pneumophila.* Subsequent computerized tomographic scans of the chest documented progressive improvement of lung infiltrates, and nearly complete resolution was obtained 3 months after hospital admission.

*L. pneumophila* SG 11 infection has, thus far, been reported only rarely in humans. The first SG 11 strain was isolated in the United States in 1982 from a patient with multiple myeloma (*4*). Since then, few other cases of SG 11 strains have been reported in Europe (*5*,*6*); it is conceivable that this strain is not as widely distributed and is less pathogenic than other SGs, especially SG 1. It can be argued that infections caused by SG 11 have been underdiagnosed. *L. pneumophila* SG 11 cannot be detected by *Legionella* urinary antigen or serologic tests, the assays most frequently used to diagnose legionellosis (*7*–*9*). The negative urinary antigen test result for this patient is consistent with a non–SG 1 infection. The single positive serologic result for IgG was probably caused by cross-reactivity because the commercial assay kit was designed to recognize only *L. pneumophila* SGs 1–7. Culture is the only useful diagnostic tool for identifying SGs. However, this tool is not always feasible because it requires specialized media and skills to identify the organism. In addition, sensitivity is low, depending on the severity of the disease and the availability of adequate respiratory specimens (*9*). Despite these limitations, culture is needed to detect all SGs of *L. pneumophila*, especially in immunocompromised patients, who are more susceptible to infections caused by strains of non–SG 1 *L. pneumophila* (*10*).
